# Supplementary material for: Effects of High Irradiance and Low Water Temperature on Photoinhibition and Repair of Photosystems in Marimo (Aegagropila linnaei) in Lake Akan, Japan
Source: Int J Mol Sci. 2022 Dec 21;24(1):60. doi: 10.3390/ijms24010060 (PMC9820325; doi:10.3390/ijms24010060)
Supplement: Supplementary file 1 [file ijms-24-00060-s001.zip › ijms-2013619-supplementary.pdf]

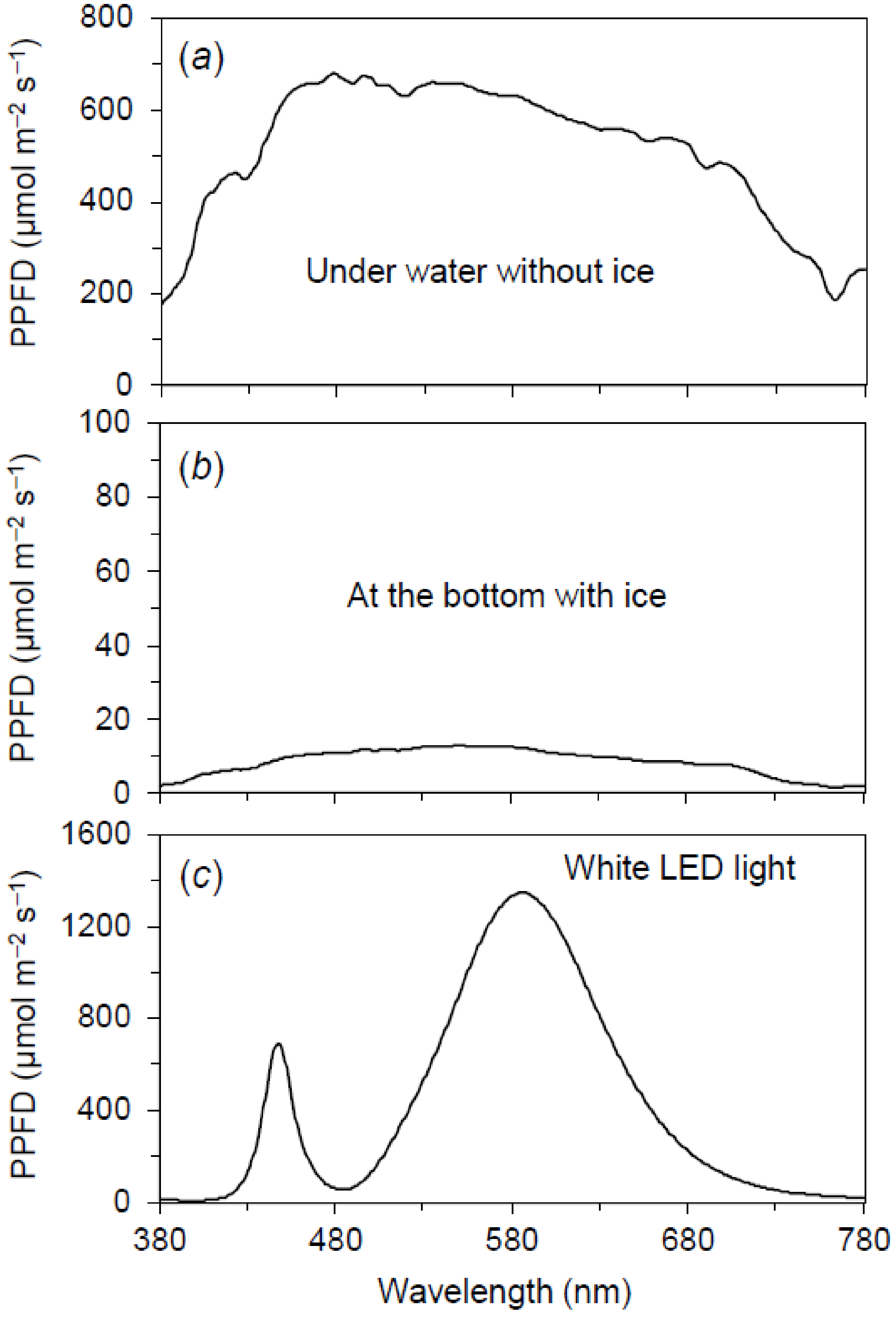

Figure S1. Spectra of the sunlight under water and the light source. Sunlight at about 30 cm in depth of water without ice (*a*) and at the bottom of 1.5 m depth with ice (*b*) in Lake Akan on sunny day, and white LEDs used for photoinhibitory and repair treatments (*c*) These spectra were measured with a handheld portable Light Analyzer LA-105 (Nippon Medical & Chemical Instruments Co., Ltd., Japan).

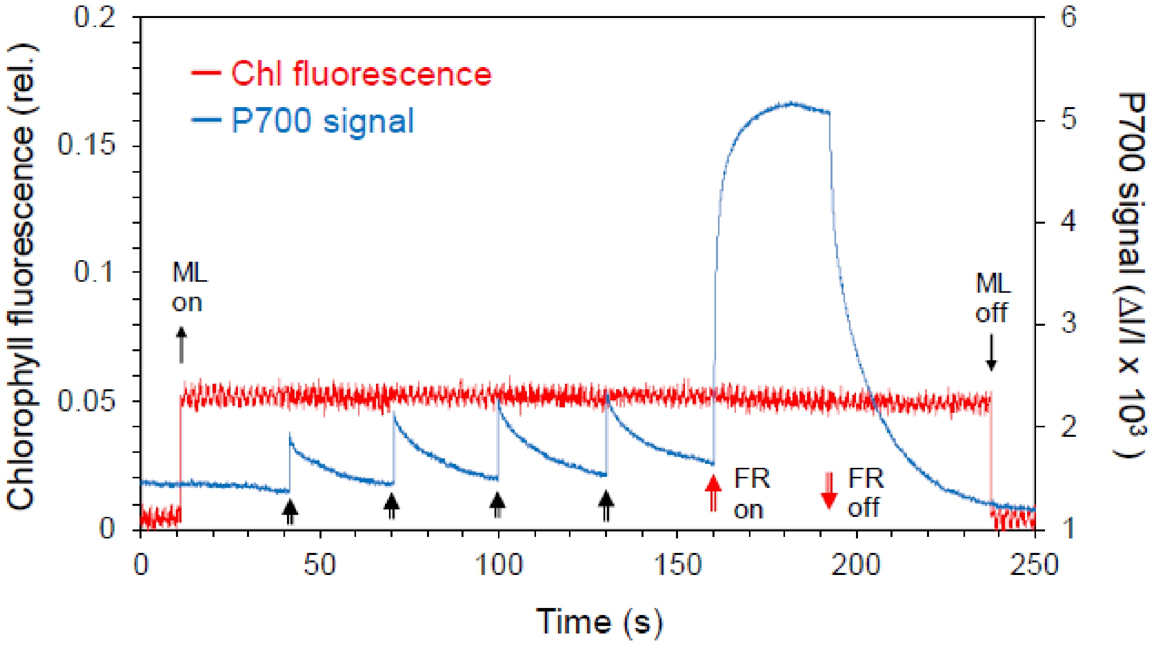

Figure S2. Changes in Chl fluorescence (red) and P700 signal (blue) in response to far-red (FR) illumination at 20.3 W m^{-2} in the Marimo filamentous cells. Bold black arrows show the applications of FR pulse for 100 ms, and bold red arrows show FR on/off. Measurements were made in the cold room (40 Pa CO_2, 21 kPa O_2, at 4˚C). ML; the measuring light for chlorophyll fluorescence.

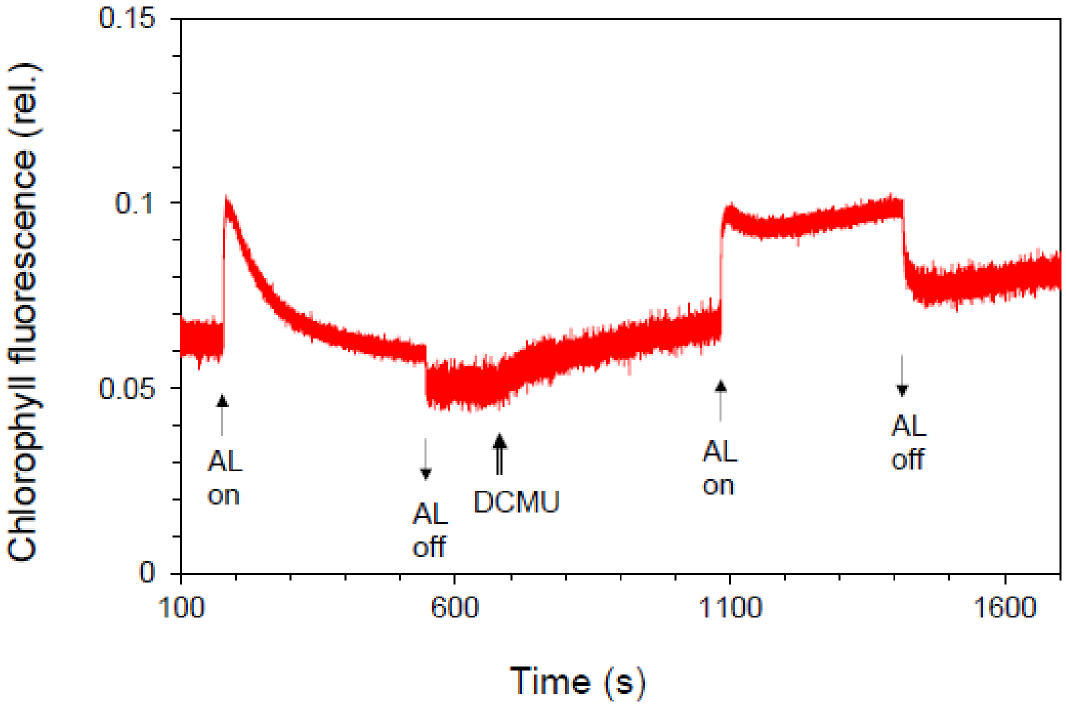

Figure S3. Changes in Chl fluorescence in response to the actinic light (AL) at 30 μmol m^{-2} s^{-1} in the absence and presence of the PSII electron transport inhibitor, DCMU, at 100 μM. Bold black arrow shows the applications of DCMU, and thin arrows show AL on/off. Measurements were made in the cold room (40 Pa CO_2, 21 kPa O_2, at 4˚C).

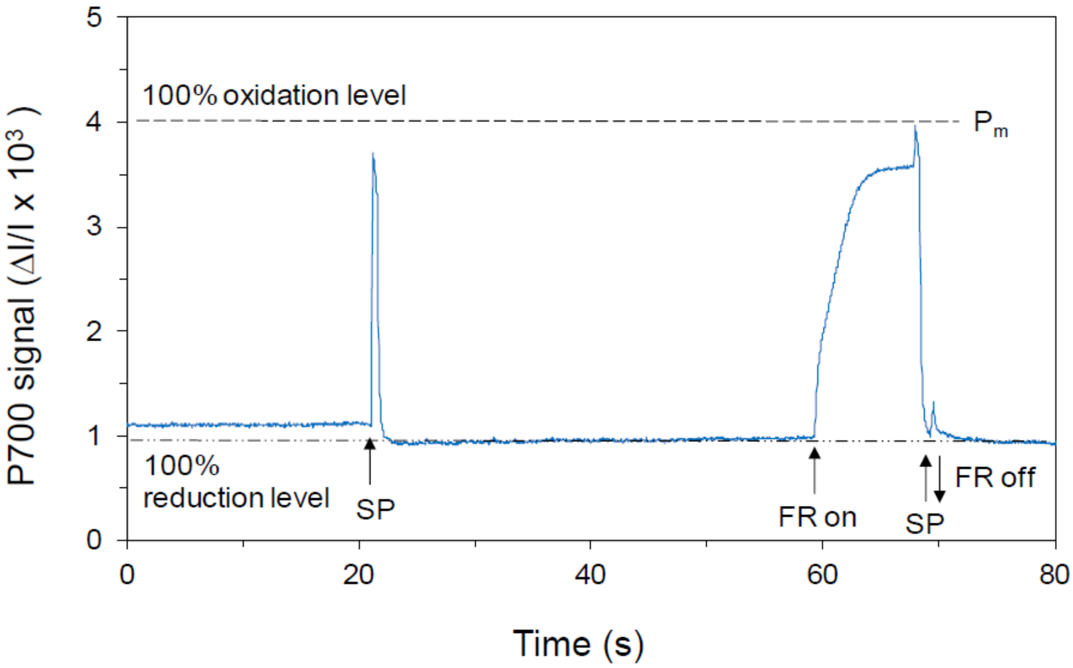

Figure S4. Changes in P700 signal in response to SP and FR illumination at 20.3 W m^{-2} in the Marimo filamentous cells without DCMU. Arrows show the applications of each illumination. Dashed line shows the levels of full oxidation (P_m) or full reduction. Measurements were made in the cold room (40 Pa CO_2, 21 kPa O_2, at 4°C). SP; saturation pulse, FR light; far-red light.

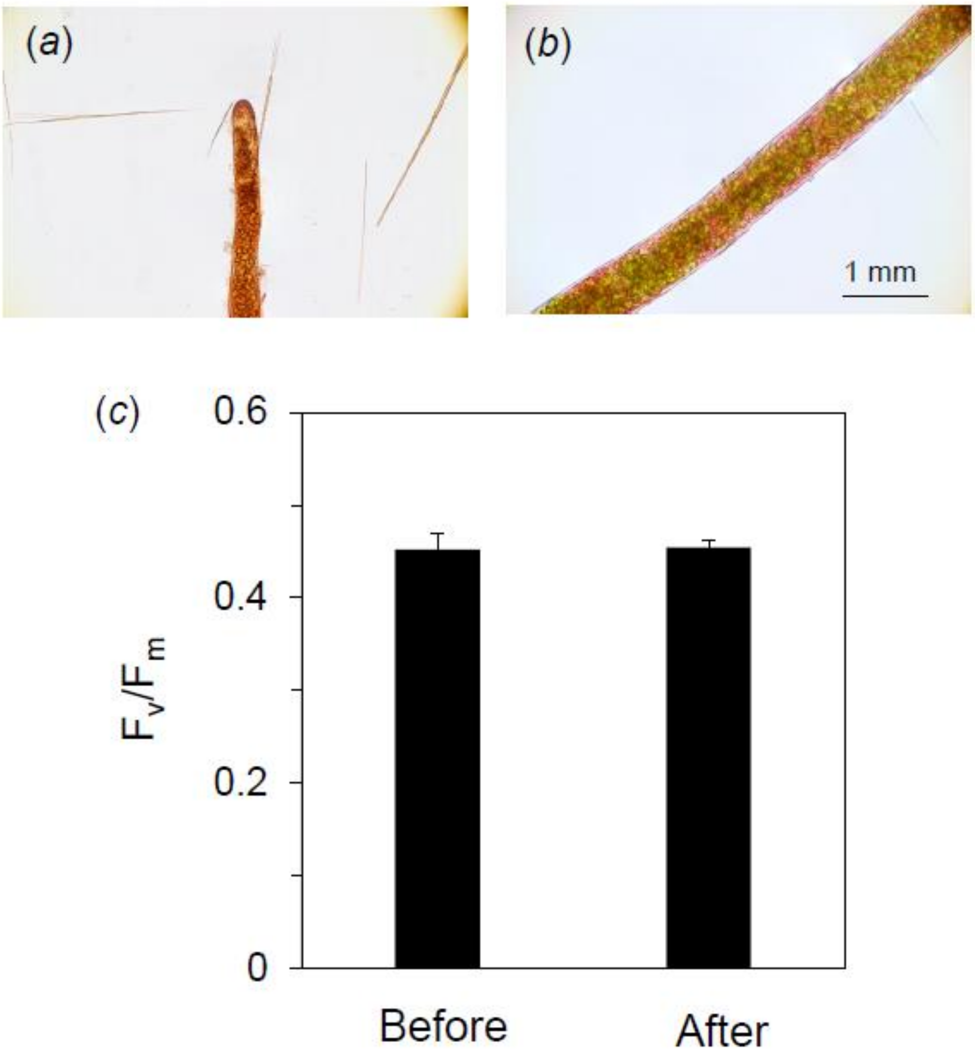

Figure S5. Neutral red uptake assay (*a* and *b*) and Fv/Fm(*c*) in the filamentous cells of Marimo before (*a*) and after the simulated light treatment for five days (*b*) at 20˚C. The cells were incubated in the growth light at 20˚C for two weeks for the acclimation, and then the measurements were done with these cells. The simulated light was same as the one used in Fig. 5. Measurements were made in the ventilated room (40 Pa CO_2, 21 kPa O_2, at 20˚C). The values represent the means ±SD (n= 3).

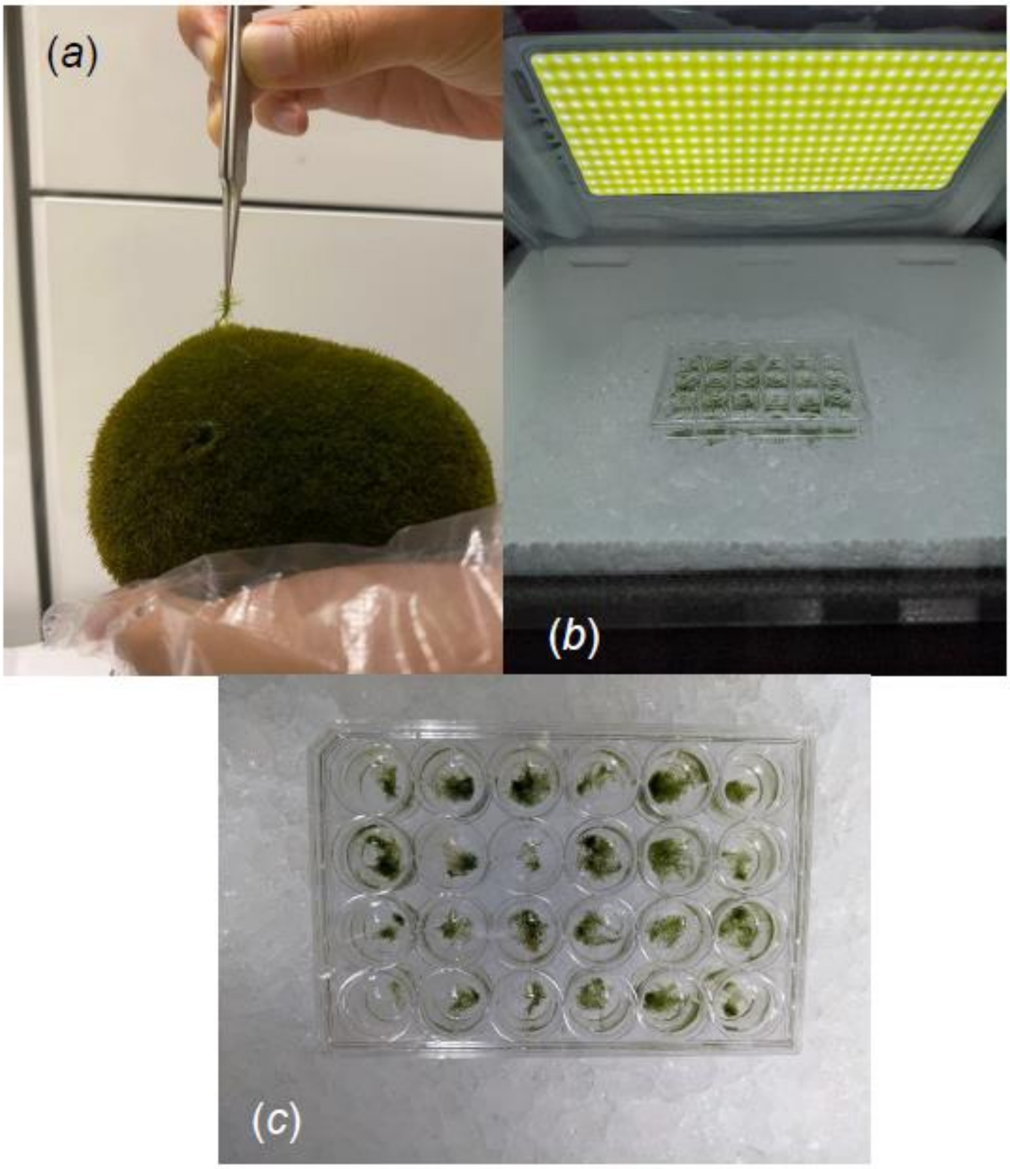

Figure S6. (*a*), Image of picking the filamentous cells of *A. linnaei* up from the surface parts of the spherical Marimo with tweezers. (*b*), Image of the photoinhibitory and repair treatments. In fact, light was provided to the filamentous cells from straight above. (*c*), Image of the dissected filamentous cells on a 24-well plate.

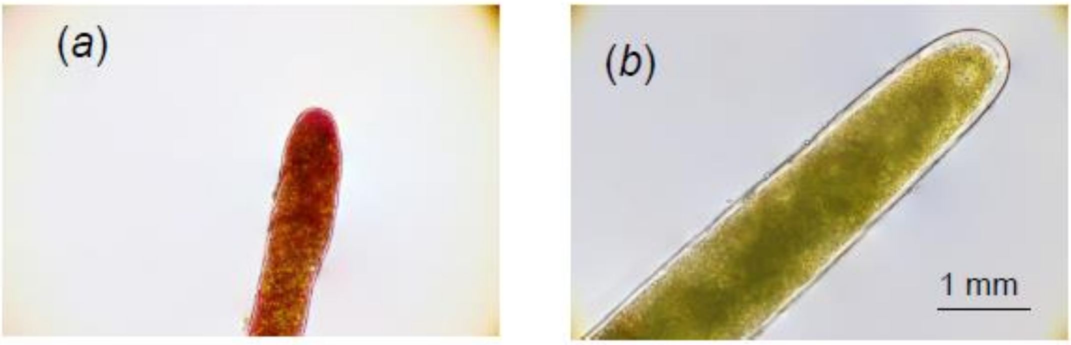

Figure S7. Neutral red uptake assay in the filamentous cells of Marimo before (a) and after the incubation at 40˚C for three days (b). The cells were incubated in the growth light at 20˚C for two weeks, and then transferred to 40˚C. The assay was done at 20˚C. (*b*) indicated that the cells died at high temperature of 40˚C.
